# Supplementary material for: Mechanisms governing the pioneering and redistribution capabilities of the non-classical pioneer PU.1
Source: Nat Commun. 2020 Jan 21;11:402. doi: 10.1038/s41467-019-13960-2 (PMC6972792; doi:10.1038/s41467-019-13960-2)
Supplement: Supplementary file 7 — Source data [file 41467_2019_13960_MOESM7_ESM.zip › Source_Data/Figure6/Figure6B_MotifScanOutput/ETS1_mutPU.1/homerResults/motif41.similar.html]

motif41

## Information for motif41

T
C
A
G
A
G
C
T
A
T
G
C
G
T
C
A
A
G
T
C
C
T
A
G
A
C
G
T
A
T
C
G
T
C
G
A
A
G
T
C
A
C
G
T
  
Reverse Opposite:  

T
G
C
A
T
C
A
G
A
G
C
T
A
T
G
C
T
G
C
A
G
A
T
C
T
C
A
G
C
A
G
T
A
T
C
G
T
C
G
A
A
G
T
C
  

|  |  |
| --- | --- |
| p-value: | 1e-37 |
| log p-value: | -8.521e+01 |
| Information Content per bp: | 1.657 |
| Number of Target Sequences with motif | 463.0 |
| Percentage of Target Sequences with motif | 3.43% |
| Number of Background Sequences with motif | 528.6 |
| Percentage of Background Sequences with motif | 1.51% |
| Average Position of motif in Targets | 97.6 +/- 54.5bp |
| Average Position of motif in Background | 97.6 +/- 59.9bp |
| Strand Bias (log2 ratio + to - strand density) | 0.0 |
| Multiplicity (# of sites on avg that occur together) | 1.05 |
| Motif File: | file (matrix) reverse opposite |

### Similar de novo motifs found

|  |  |  |  |  |  |  |  |
| --- | --- | --- | --- | --- | --- | --- | --- |
| Rank | Match Score | Redundant Motif | P-value | log P-value | % of Targets | % of Background | Motif file |
| 1 | 0.988 | T C A G A G C T T A G C G C T A A G T C T C A G C A G T C T A G C T G A A T G C A G T C A T G C | 1e-32 | -75.788823 | 2.83% | 1.20% | motif file (matrix) |
| 2 | 0.958 | T A G C T C A G T C A G T A C G G A C T A T G C T G C A A G T C T C A G A C G T A T C G T C G A A G T C | 1e-32 | -74.192183 | 2.96% | 1.29% | motif file (matrix) |
